# Frequency and indication of non-musculoskeletal examinations: a cross-sectional survey of Quebec chiropractors

**DOI:** 10.1186/s12998-023-00522-z

**Published:** 2024-02-28

**Authors:** Danikel Giroux, Chloé Branconnier, André Bussières, Jean Théroux, Marc-André Blanchette

**Affiliations:** 1 Departement de Chiropratique, Université du Québec à Trois-Rivières (UQTR), Trois-Rivières, QC Canada; 2 School of Physical and Occupational Therapy, Faculty of Medicine, McGill University, Montreal, QC Canada; 3 School of Allied Health, Chiropractic Discipline, Murdoch University, Perth, WA Australia

**Keywords:** Triage, Physical examinations, Chiropractic, Diagnostic tests, Routine, Diagnostic techniques and procedures

## Abstract

**Background:**

Approximately 1% of low back pain is estimated to be caused by serious systemic diseases, including cancer, infection, or abdominal aortic dissection. This study aimed to determine the frequency of execution of non-MSK physical examination procedures among Quebec chiropractors and to identify the clinical context that prompts them to use these physical examination procedures.

**Methods:**

Cross-sectional survey containing 44 questions administered to a random sample of Quebec chiropractors using a succession of online, postal and phone questionnaires. The 4-part survey questionnaire contained six demographic questions, 28 single-choice questions to determine the frequency of execution of non-MSK physical examination procedures, seven short clinical vignettes for which the respondents had to select the non-MSK examinations that would be required, and two questions inquiring about the proportion of new patients for which participants’ felt non-MSK examinations were necessary and whether appropriate assessments were performed. The questionnaire was pilot tested, and feedback received integrated prior to administration. We conducted descriptive statistics, Pearson correlations, and an ANOVA.

**Results:**

The survey was completed by 182 chiropractors (response rate: 36.4%). The most commonly non-musculoskeletal examination performed daily were blood pressure (12.1%) and cranial nerves (4.9%). The most common tests never performed were oxygen saturation (68.7%), cardiac auscultation (69.2%), tibio-brachial index (71.4%), breast (86.8%), rectal (96.7%), testicular (95.6%), and vaginal (99.9%) exams. Female chiropractors and Quebec University in Trois-Rivières graduates reported that a significantly higher proportion of their new patients required a non-musculoskeletal physical examination compared to male participants (37.2% vs 28.3%) or Canadian Memorial Chiropractic College graduates (33.9% vs 19.9%). Reason for not performing a physical examination included the belief that another healthcare professional was better positioned to perform and/or interpret the related tests (76.4%).

**Conclusions:**

Vital signs and cranial nerve examinations were the most frequency performed non-musculoskeletal examinations reported by chiropractors. Apart from the genitourinary exam almost never performed, most participants chose non-musculoskeletal examinations deemed appropriate for the patient’s presentation.

**Supplementary Information:**

The online version contains supplementary material available at 10.1186/s12998-023-00522-z.

## Background

Chiropractors are primary care providers and therefore they most determine themselves if chiropractic care is indicated for their patient using patient interview and physical examination. The most frequently reported reasons for attending chiropractic care are low back pain (49.7%), neck pain (22.5%), limb pain (10.0%) and headaches (5.5%) [1]. While the majority of these complaints are caused by musculoskeletal conditions, non-musculoskeletal (non-MSK) pathologies can initially present with similar symptoms [2, 3]. For example, 65.0% of renal colic patients have low back pain (flank pain) [4], 14.9% of ischemic stroke patients experience headaches at onset [5] and more than haft of chest pain (50% to 66%) is not of musculoskeletal origin (e.g. pleurisy, pneumonia, angina, esophagitis) [6, 7]. Approximately 1% of low back pain is estimated to be caused by serious systemic diseases, including cancer, infection, or abdominal aortic dissection [8].

As primary healthcare providers, chiropractors must determine whether their patients’ conditions fall within their scope of practice focusing on musculoskeletal disorders [9]. The patient history and physical examination inform diagnostic probabilities, helping guide decision-making regarding subsequent clinical management [10]. Although physical examination findings contribute only to about 12% of the diagnosis, it often allows to exclude hypotheses raised during the patient’s history and increases the professionals' confidence in their diagnoses [11, 12]. The absence of any indicator of serious pathology and a normal physical examination commonly suggests acute or chronic non-specific low back pain for which manual therapy is generally recommended in clinical practice guidelines [13]. Conversely, a suspicion of underlying serious conditions with positive physical examination findings (e.g. sudden onset of low back pain radiating to the iliac fossa and increased renal percussion suggesting renal colic) require prompt medical referral for further investigations and adequate management [14, 15].

Clinical practice guidelines on the management of musculoskeletal conditions emphasize the need to rule out serious underlying pathologies before managing a patient. This aspect is central to the chiropractic legislation and academic curriculum in Quebec [9, 16]. However, there is no clear guidance on how to rule out specific non-MSK conditions [2, 3, 17]. Consequently, the teaching and practice standards regarding the use of non-MSK physical examination procedures are uncertain [18]. Furthermore, little is known about the frequency at which chiropractors perform non-MSK examinations and the clinical indications that prompt them to perform these examinations. As a first step towards better defining what examination procedure should be done by chiropractors, we begin by assessing what is currently done. Thus, this study aimed (1) to determine the frequency of execution of non-MSK physical examination procedures among Quebec chiropractors and (2) to identify the clinical context that prompts them to use these physical examination procedures.

## Methods

### Study design

We administered a cross-sectional survey to Quebec chiropractors using an online questionnaire.

### Population and eligibility criteria

To be eligible for the study, participants had to hold a chiropractic degree and a valid license to practice chiropractic in the province of Quebec [member of l’*Ordre des chiropraticiens du Québec* (OCQ)], do a minimum of ten (10) hours of clinical work per week [19], and/or be a lecturer, professor or clinician at the chiropractic program at l’Université du Québec à Trois-Rivières (UQTR).

### Data collection

All participants were recruited between August 2021 and February 2022. Among the 1286 OCQ members with publicly available contact information (www.ordredeschiropraticiens.ca) in 2021, 450 chiropractors were randomly selected using SPSS.v26 IBM Corporation, Armonk, NY. This sample size was based on a 7.5% margin of error while accounting for an expected response rate of 33%. In addition, the invitation to complete the survey was also sent to all the 50 UQTR-affiliated chiropractic educators.

Selected chiropractors were invited to complete the online survey. Email reminders were sent every two weeks for six weeks. Participants who had not responded to the survey by the end of the six weeks were contacted by mail, and the remaining non-responders were contacted by phone [20]. Before completing the survey, eligible chiropractors were invited to read the study information and consent to participate by clicking the “agree” button.

### Instrument

The online survey was anonymous and administered via the « *Banque interactive de question*» (BIQ), a survey tool created by the *Université du Québec à Trois-Rivières* (https://confluence.uqtr.ca/display/AOPSP/BIQ). Our questionnaire was purposely developed for this study and included four main sections (Additional file 1). The first section comprised six questions about the respondents’ demographics (gender, number of years of practice, school they graduated from, etc.) enabling us to verify their eligibility. The second section included 28 single-choice questions to determine the frequency of execution of non-MSK physical examination procedures (at least once a day, at least once a week, at least once a month, at least once a trimester, at least once a year, never). The third section presented seven short clinical vignettes for which the respondents had to select all the non-MSK examinations (listed in the second section) that would be required (multiple-choice questions). The clinical vignettes were adapted from those presented in a recent textbook on the clinical approach [21]. The two chiropractic interns (CB, DG) developed a first draft of seven clinical vignettes, which were subsequently revised by two faculty members (AB, MAB) teaching non-MSK examinations at the UQTR chiropractic program. The fourth section contained two free text questions, one inquiring about the proportion of new patients for which participants’ felt non-MSK examinations were necessary and whether appropriate assessments were performed and the second investigated reasons for not performing non-MSK examinations using multiple-choice answers. The questionnaire was first pilot tested among five chiropractic interns and five academic teachers, asking respondents about the questionnaire's exhaustivity and clarity on a 1 to 4 scale. Minor changes were made based on feedback obtained to help clarify survey items.

### Data analysis

All variables collected were analyzed descriptively (frequency, percentages, mean, standard deviation). We conducted Pearson correlation (continuous variables) to assess the association between the respondent characteristics, the percentage of their new patient for which a non-MSK procedure would be required, and the percentage of these patients for which the chiropractors would perform the required non-MSK examination. We conducted ANOVA (categorical variables) to compare the means between the respondent characteristics for the percentage of their new patient for which a non-MSK procedure would be required, and the percentage of these patients for which the chiropractors would perform the required non-MSK examination. Fischer's least significant difference post hoc test was conducted when appropriate [22]. Our a priori hypotheses were that men, more experienced clinicians, chiropractors trained outside of Canada and without postgraduate studies would perform less non-MSK examination. All analyses were completed in SPSSv26.0 (IBM Corporation, Armonk, NY). A *p*-value ≤ 0.05 was used as the level of statistical significance.

### Ethical considerations

This research project was approved by the UQTR Research Ethics Board (CER-21–276-07.16).

## Results

**Fig. 1:**
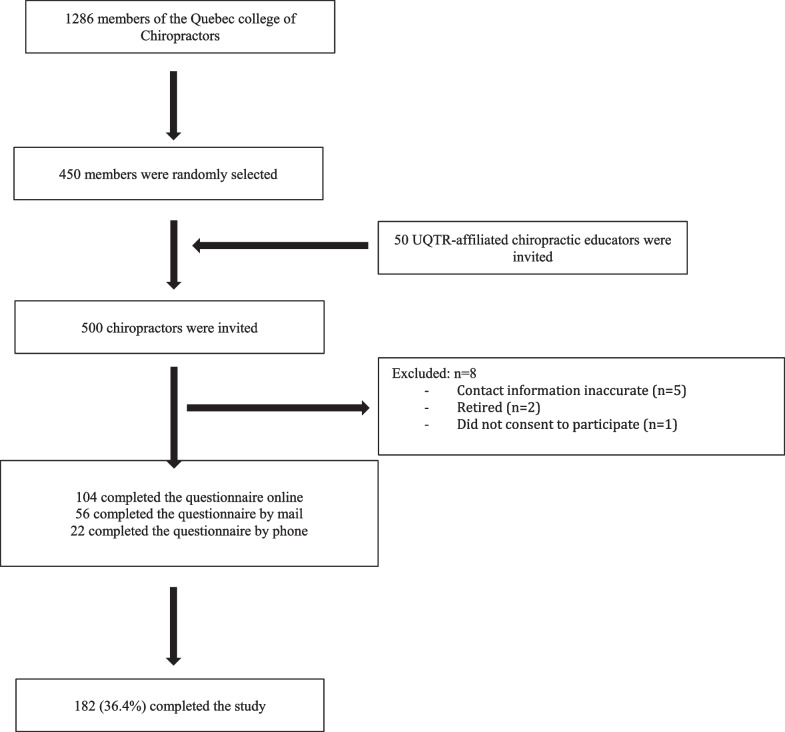
Flow chart diagram showing the inclusion and exclusion of respondents through each stage of the study

Among the 450 chiropractors solicited, eight were excluded because of the inaccuracy of their contact information (n = 5), they had retired (n = 2), or they did not consent to participate (n = 1). One hundred eighty-two eligible chiropractors completed the questionnaire leading to a response rate of 36.4% (Fig. 1).

The respondents’ characteristics are presented in Table 1. Most respondents were women (52.7%) who graduated from UQTR (72.5%). The mean number of hours worked per week was 29.7 h, and the mean number of years of practice was 17.1 years. Our sample was not significantly different than licensed chiropractors in Quebec for gender (women: 52.7% vs. 46.0%, *p* = 0.08), and number of years of experience (17.1 years vs. 16 years, *p* = 0.22) using chi^2^ and t-test respectively [23].

**Table 1:** Demographics of the respondents (n = 182)

Characteristics of the respondents		n (%)
Gender	Male	84 (46.2)
	Female	96 (52.7)
	Missing	2 (1.1)
Educational institution for chiropractic training	UQTR	132 (72.5)
	CMCC	24 (13.2)
	Other	26 (14.3)
Post-graduate chiropractic studies	None	129 (70.9)
	MSc/PhD	22 (12.1)
	Specialty/fellow/diplomate	26 (14.3)
	MSc/PhD and Specialty/fellow/diplomate	5 (2.7)
Employed in a Canadian chiropractic educational institution	None	159 (87.4)
	Lecturer	12 (6.6)
	Professor	3 (1.6)
	Clinician	5 (2.7)
	Missing	3 (1.6)

		Mean (SD)
Number of chiropractic working hours per week		29.7 (8.9)
Number of years of practice in the chiropractic profession		17.1 (11.8)

*SD* Standard Deviation; *UQTR* Université du Québec à Trois-Rivières; *CMCC* Canadian Memorial Chiropractic College

The most common non-MSK assessments were blood pressure performed at least once a day (12.1% of participants), and cranial nerves examination performed at least once a week (36.3%). More than half of the participants reported never performing the following procedures: temperature (58.2%), oxygen saturation (68.7%), fundus observation (57.7%), pulmonary auscultation (56.6%), cardiac auscultation (69.2%), arterial auscultation (56.6%), tibio-brachial index (71.4%), breast examination (86.8%), digital rectal exam (96.7%), vaginal exam (98.2%) and testicular exam (95.6%) (Table 2).

**Table 2:** Frequency of performing physical examination procedures reported by the respondents

		At least once a day	At least once a week	At least once a month	At least once a trimester	At least once a year	Never	Missing
Vital signs	Blood pressure	22 (12.1%)	33 (18.1%)	**49 (26.9%)**	43 (23.9%)	20 (11.0%)	13 (7.1%)	2 (1.1%)
	Cardiac frequency	18 (9.9%)	24 (13.2%)	**49 (26.9%)**	30 (16.5%)	24 (13.2%)	35 (19.2%)	2 (1.1%)
	Respiratory frequency	7 (3.8%)	15 (8.2%)	27 (14.8%)	24 (13.2%)	31 (17.0%)	**74 (40.7%)**	4 (2.2%)
	Temperature	13 (7.1%)	1 (0.5%)	7 (3.8%)	21 (11.5%)	32 (17.6%)	**106 (58.2%)**	2 (1.1%)
	Saturation	10 (5.5%)	13 (7.1%)	5 (2.7%)	17 (9.3%)	10 (5.5%)	**125 (68.7%)**	2 (1.1%)
Neurological examination	Cranial nerves	9 (4.9%)	**66 (36.3%)**	56 (30.8%)	27 (14.8%)	16 (8.8%)	5 (2.7%)	3 (1.6%)
	Vestibular manouver	7 (3.8%)	22 (12.1%)	**70 (38.5%)**	60 (33.0%)	11 (6.0%)	8 (4.4%)	4 (2.2%)
	Cerebellar function	10 (5.5%)	38 (20.9%)	**52 (28.6%)**	41 (22.5%)	25 (13.7%)	14 (7.7%)	2 (1.1%)
	Meningeal irritation signs	9 (4.9%)	22 (12.1%)	35 (19.2%)	**51 (28.0%)**	40 (22.0%)	23 (12.6%)	2 (1.1%)
Ophthalmic examination	Observation of the eye fundus	0 (0.0%)	0 (0.0%)	11 (6.0%)	14 (7.7%)	49 (26.9%)	**105 (57.7%)**	3 (1.6%)
Otorhinolaryngologic examination	Observation of the eardrum	5 (2.7%)	32 (17.6%)	**54 (29.7%)**	44 (24.2%)	21 (11.5%)	24 (13.2%)	2 (1.1%)
Pulmonary examination	Pulmonary auscultation	0 (0.0%)	4 (2.2%)	17 (9.3%)	24 (13.2%)	32 (17.6%)	**103 (56.6%)**	2 (1.1%)
Cardiac examination	Cardiac auscultation	0 (0.0%)	5 (2.7%)	9 (4.9%)	15 (8.2%)	25 (13.7%)	**126 (69.2%)**	2 (1.1%)
Peripheral vascular examination	Arteries auscultation	2 (1.1%)	6 (3.3%)	11 (6.0%)	20 (11.0%)	39 (21.4%)	**102 (56.0%)**	2 (1.1%)
	Tibio-brachial index	0 (0.0%)	2 (1.1%)	3 (1.6%)	15 (8.2%)	28 (15.4%)	**130 (71.4%)**	4 (2.2%)
	Allen test	4 (2.2%)	12 (6.6%)	25 (13.7%)	50 (27.5%)	37 (20.3%)	**51 (28.0%)**	3 (1.6%)
	Homan	4 (2.2%)	10 (5.5%)	21 (11.5%)	**68 (37.4%)**	45 (24.7%)	32 (17.6%)	2 (1.1%)
	Godet's sign	9 (4.9%)	16 (8.8%)	45 (24.7%)	**68 (37.4%)**	24 (13.2%)	17 (9.3%)	3 (1.6%)
	Buerger/Tredelenberg	3 (1.6%)	10 (5.5%)	10 (5.5%)	27 (14.8%)	34 (18.7%)	**96 (52.7%)**	2 (1.1%)
	Limb circumference	1 (0.5%)	6 (3.3%)	12 (6.6%)	46 (25.3%)	**60 (33.0%)**	55 (30.2%)	2 (1.1%)
Abdominal examination	Abdomen general exam	3 (1.6%)	25 (13.7%)	32 (17.6%)	43 (23.6%)	**46 (25.3%)**	31 (17.0%)	2 (1.1%)
	Abdominal specific organ palpation	2 (1.1%)	17 (9.3%)	34 (18.7%)	34 (18.7%)	38 (20.9%)	**54 (29.7%)**	3 (1.6%)
	Special maneuver (e.g. Kidney punch)	4 (2.2%)	13 (7.1%)	34 (18.7%)	51 (28.0%)	**55 (30.2%)**	23 (12.6%)	2 (1.1%)
Breast examination	Breast exam	0 (0.0%)	0 (0.0%)	4 (2.2%)	3 (1.6%)	15 (8.2%)	**158 (86.8%)**	2 (1.1%)
Genito-urinary examination	Rectal exam	0 (0.0%)	0 (0.0%)	0 (0.0%)	1 (0.5%)	3 (1.6%)	**176 (96.7%)**	2 (1.1%)
	Vaginal exam	0 (0.0%)	0 (0.0%)	0 (0.0%)	0 (0.0%)	0 (0.0%)	**180 (98.9%)**	2 (1.1%)
	Testicular exam	0 (0.0%)	0 (0.0%)	0 (0.0%)	0 (0.0%)	5 (2.7%)	**174 (95.6%)**	3 (1.6%)
	Inguinal canal exam	0 (0.0%)	3 (1.6%)	8 (4.4%)	42 (23.1%)	48 (26.4%)	**79 (43.4%)**	2 (1.1%)

Bold: mode

Except for vignette 1 (low back and testicular pain), vital signs commonly selected by participants across other vignettes were blood pressure and cardiac frequency. In contrast, respiratory frequency was assessed by most respondents in presence of chest pain only (vignettes 3 and 4). The oxygen saturation was considered with chest pain exacerbated by activity (vignette 3), but not with shortness of breath (vignette 4). In most clinical contexts, the body temperature was the least considered. Most participants included pulmonary and cardiac auscultation when presented with chest pain (vignettes 3 and 4). Similarly, the ophthalmoscopic and cranial nerve exams were selected when presented with a patient with visual symptoms (vignette 5). More than 80% of participants indicated that performing cranial nerves, blood pressure, and vestibular examination in patients with headaches, vertigo, hearing loss, and ear pressure (vignette 7) was appropriate. For low back pain irradiating to the groin and testicular region (vignette 1), most respondents indicated they would perform an inguinal canal examination but not abdominal and testicular assessments. Most respondents would have verified the blood pressure when presented with a clinical context suggesting mechanical neck pain (vignette 6) (Table 3).

**Table 3:** Physical examination procedure selected by the respondent for seven clinical vignettes

	Vignette 1 n (%)	Vignette 2 n (%)	Vignette 3 n (%)	Vignette 4 n (%)	Vignette 5 n (%)	Vignette 6 n (%)	Vignette 7 n (%)
	45 year old male presenting with acute LBP irradiating in left groin and testicle	65 year old overweight male smoking, suffering from HPB, hyperlipidemia and presenting with acute LBP	76 year old male presenting with acute right chest pain relieved at rest and worsened with activity	22 year old stressed female complaining with shortness of breath and acute right chest pain	26 year old female complaining of floaters in left eye after it being hit	43 year old female presenting with acute left neck pain now migrating to the right and preventing her from turning head	68 year old overweight male complaining with morning headaches since a month and acute vertigos, hearing loss and ear pressure
Blood pressure	85 (46.7%)	**138 (75.8%)**	**164 (90.1%)**	**142 (78.0%)**	63 (34.6%)	**102 (56.0%)**	**147 (80.8)**
Cardiac frequency	48 (26.4%)	**106 (58.2%)**	**163 (89.6%)**	**142 (78.0%)**	37 (20.3%)	77 (42.3%)	**106 (58.2%)**
Respiratory frequency	23 (12.6%)	68 (37.4%)	**128 (70.3%)**	**144 (79.1%)**	27 (14.8%)	52 (28.6%)	76 (41.8%)
Temperature	48 (26.4%)	34 (18.7%)	36 (19.8%)	40 (22.0%)	16 (8.8%)	40 (22.0%)	30 (16.5%)
Saturation	19 (10.4%)	54 (29.7%)	**94 (51.6%)**	86 (47.3%)	16 (8.8%)	24 (13.2%)	51 (28.0%)
Cranial nerves	7 (3.8%)	3 (1.7%)	7 (3.8%)	7 (3.8%)	**139 (76.4%)**	76 (41.8%)	**149 (81.9%)**
Vestibular maneuver	3 (1.7%)	2 (1.1%)	3 (1.65%)	2 (1.1%)	49 (27.0%)	3 (1.7%)	**158 (86.8%)**
Cerebellar function	5 (2.8%)	5 (2.8%)	7 (3.8%)	4 (2.2%)	55 (30.2%)	4 (2.2%)	**117 (64.3%)**
Meningeal irritation signs	4 (2.2%)	8 (4.4%)	3 (1.65%)	5 (2.8%)	24 (13.2%)	74 (40.7%)	61 (33.5%)
Ophthalmoscopic exam	1 (0.5%)	4 (2.2%)	7 (3.8%)	3 (1.7%)	**148 (81.3%)**	10 (5.5%)	16 (8.8%)
Otoscopic exam	0 (0%)	0 (0%)	0 (0%)	0 (0%)	2 (1.1%)	1 (0.5%)	**125 (68.7%)**
Pulmonary auscultation	5 (2.8%)	33 (18.1%)	**114 (62.6%)**	**124 (68.1%)**	3 (1.7%)	20 (11.0%)	28 (15.4%)
Cardiac auscultation	5 (2.8%)	35 (19.2%)	**138 (75.8%)**	**93 (51.1%)**	2 (1.1%)	18 (9.9%)	31 (17.0%)
Arteries auscultation	45 (24.7%)	**94 (51.6%)**	61 (33.5%)	33 (18.1%)	7 (3.8%)	41 (22.5%)	58 (31.9%)
Tibio-brachial index	10 (5.5%)	18 (9.9%)	7 (3.8%)	2 (1.1%)	0 (0%)	1 (0.5%)	2 (1.1%)
Allen test	2 (1.1%)	1 (0.5%)	6 (3.3%)	6 (3.3%)	0 (0%)	5 (2.8%)	4 (2.2%)
Homan	7 (3.8%)	7 (3.8%)	0 (0%)	7 (3.8%)	0 (0%)	0 (0%)	0 (0%)
Godet's sign	20 (11.0%)	35 (19.2%)	14 (7.7%)	6 (3.3%)	0 (0%)	1 (0.5%)	3 (1.7%)
Buerger/Tredelenberg	16 (8.8%)	21 (11.5%)	8 (4.4%)	5 (2.8%)	2 (1.1%)	3 (1.7%)	4 (2.2%)
Circonference	16 (8.8%)	19 (10.4%)	3 (1.7%)	3 (1.7%)	0 (0%)	0 (0%)	0 (0%)
Abdomen general exam	89 (48.9%)	**109 (59.9%)**	15 (8.2%)	11 (6.0%)	0 (0%)	0 (0%)	0 (0%)
Abdominal specific organ palpation	72 (39.6%)	77 (42.3%)	5 (2.8%)	5 (2.8%)	0 (0%)	0 (0%)	0 (0%)
Special maneuver (e.g. Kidney punch)	90 (49.5%)	**99 (54.4%)**	4 (2.2%)	2 (1.1%)	0 (0%)	0 (0%)	2 (1.1%)
Breast exam	0 (0%)	0 (0%)	3 (1.7%)	5 (2.8%)	0 (0%)	0 (0%)	0 (0%)
Rectal exam	7 (3.8%)	8 (4.4%)	0 (0%)	0 (0%)	0 (0%)	0 (0%)	0 (0%)
Vaginal exam	0 (0%)	0 (0%)	0 (0%)	0 (0%)	0 (0%)	0 (0%)	0 (0%)
Testicular exam	57 (31.3%)	1 (0.5%)	0 (0%)	0 (0%)	0 (0%)	0 (0%)	0 (0%)
Inguinal canal exam	**131 (72.0%)**	9 (4.9%)	2 (1.1%)	0 (0%)	0 (0%)	0 (0%)	0 (0%)

HBP: High blood pressure

LBP: Low back pain

Bold: mode

Respondents reported that approximately one third (32.7%) of their new patients required a non-MSK examination. Nonetheless, chiropractors conducted a non-MSK examination in only 58.8% of them. In contrast, female participants and those who graduate from UQTR reported a significantly higher proportion of new patients requiring a non-MSK physical examination. Nonetheless, there were no significant differences for gender or school of graduation for performing a non-MSK exam when needed on a new patient (Table 4).

**Table 4:** Association between the respondent characteristics and the percentage of new patients requiring a non-musculoskeletal examination and the percentage of these patients for which the chiropractors performed the non-musculoskeletal examination

Respondents’ characteristics		% of new patients needing a N-MSK examination		% of patients requiring a N-MSK examination for which the respondents performed the N-MSK examination	
		Mean (SD)	*p*-value	Mean (SD)	*p*-value
Complete sample		32.7 (28.1)	–	58.8 (36.1)	–
Gender	Men	28.3% (26.4)	0.043	60.5% (34.4)	0.57
	Women	37.2% (29.1)		57.3% (37.8)	
Educational institution for chiropractic training	UQTR	33.9% (26.6)	0.045	57.0% (35.3)	0.29
	CMCC	19.9% (21.6)^1^		56.4% (40.0)	
	Other	39.0% (36.9)		69.6% (36.1)	
Post-graduate chiropractic studies	None	32.4% (27.7)	0.99	62.4% (35.1)	0.09
	MSc/PhD	34.5% (29.4)		41.1% (36.1)	
	Specialty/fellow/diplomate	32.3% (28.4)		57.1% (38.8)	

		r	*p*-value	r	*p*-value
Number of chiropractic working hours per week	0.03	0.71	0.01	0.86	
Number of years of practice in the chiropractic profession	− 0.09	0.25	− 0.03	0.75	

^1^Significantly less for UQTR and other educational institutions; r:Pearson correlation coefficient; N-NSK: non-musculoskeletal; SD: Standard deviation

Among all the different reasons for not performing a non-MSK evaluation when recommended, the belief that another healthcare professional was better positioned to perform and/or interpret related tests (76.4%) and that this type of examination was beyond their scope of competence (4.4%) was most commonly reported (Table 5).

**Table 5:** Reasons* selected by the respondents for not performing a non-musculoskeletal physical examination (n = 182)

	n (%)
I consider that I will not get any information relevant to my clinical management from this examination	21 (11.5%)
I consider performing and/or interpreting non-musculoskeletal physical examinations to be beyond my current competence	47 (25.8%)
I consider that another health care professional will be in a better position to perform and/or interpret the information from this physical examination than a chiropractor	139 (76.4%)
I consider non-musculoskeletal examinations to be outside my scope of practice	8 (4.4%)
I don't have time	6 (3.3%)
Other reasons	32 (17.6%)

*Respondent might select more than one reason

## Discussion

Patients seeking care for a musculoskeletal complaint should be screened to identify those with a higher likelihood of serious underlying pathology [17]. Non-MSK examination can help establish patients’ management and prognosis or identify those requiring referrals to another practitioner for further evaluation [10]. Several non-musculoskeletal differential diagnoses were plausible based on the parsimonious clinical information provided in the seven vignettes. The most frequently performed non-MSK exams were vital signs, including blood pressure and heart rate, and cranial nerves examination. In contrast, the least performed examination concerned the genitourinary system. Believing that another healthcare provider should perform the non-musculoskeletal evaluation was the main reason provided by chiropractors for not doing a non-musculoskeletal assessment. Interestingly, female chiropractors and UQTR graduates were more likely to report performing non-MSK assessments for their new patients.

An estimated 3.1% of adult chiropractic patients seek care for a non-musculoskeletal condition, and that proportion is higher among paediatric patients [1]. A random sample survey of Canada's English-speaking chiropractors found that 95% of respondents performed some form of differential diagnosis with their new patients [24]. Although the study mainly focused on the standard clinical assessments for musculoskeletal conditions (history taking, range of motion, orthopedic and neurological exams, manual palpation), 28.5% of the respondents commonly took the blood pressure on their new patients' [24]. Similarly, less than one third of our study participants reported measuring blood pressure once a month. Complete vital signs assessment is often recommended in the presence of chest pain in the primary care setting [25]. Our study participants frequently selected respiratory frequency in the presence of chest pain and blood pressure and heart rate when cardiovascular risk factors were mentioned, such as chest pain, headaches, and vertigo. Saturation was not uniformly requested in the presence of cardiorespiratory symptoms, and most participants did not plan to assess the patient's temperature in any of the selected clinical contexts.

When considering an older man with back pain and cardiovascular risk factors (vignette 2), most respondents choose to perform an abdominal examination, including vascular auscultation, possibly to assess the eventuality of an abdominal aortic aneurysm. These procedures are commonly used to diagnose abdominal aortic aneurysms, despite their poor sensitivity [26]. When suspected, clinicians should request an ultrasound examination, as abdominal aortic aneurysms cannot be ruled out based only on the clinical examination.

Most of our respondents chose to examine the area related to the symptoms presented (pulmonary and cardiac assessment for chest pain; cranial nerves and otoscopic exam for eye complaints, vestibular maneuver and cerebellar function for vertigo; otoscopic exam for ear symptoms; and cranial nerves for headache). Unfortunately, most respondents omitted to assess vital signs or examine the abdomen and the testicle in a vignette of a male patient presenting with low back pain radiating to the groin and testicle. These procedures might be useful to identify other genitourinary conditions such as renal colic, pyelonephritis and sexually transmissible disease [27]. A similar reluctance to inquire about genitourinary symptoms and perform related physical examination procedures has been observed among other healthcare providers [28].

The general physician utilization rate of specific diagnostic tests appears to be influenced by the ease of performance, time constraints, and patient expectations [29]. Since chiropractors do not have access to laboratory testing or advanced imaging in many jurisdictions, the physical examination is even more critical to inform the diagnosis and clinical management. The public desires and expects a thorough physical examination and history from their treating clinicians [29]. Increased use of pertinent non-musculoskeletal exam procedures by chiropractors could help improve the public and other healthcare providers' confidence in their ability to determine whether a condition is within their scope of practice, benefiting the public healthcare system from a triage perspective.

Now that the frequency of use has been determined for non-MSK physical examination procedures, future work in this field should determine standards of teaching and practice by considering the scientific evidence, the healthcare resources, and the legal implications.

### Limitations

While we obtained a response rate of 36.4% which is not uncommon among healthcare providers [30], it raises the possibility of selection bias. Although we did not find significant differences between respondents' and non-respondents’ gender and experience, it is conceivable that the respondents might differ from the non-respondents.

Since we initially wish to compare the chiropractic educators to the non-educators, we have specifically invited all the UQTR affiliated chiropractic educators. Too few educators have completed our survey to perform comparative analysis, but they are still overrepresented within our sample (10.9% in our sample vs. 3.9% of the population).

Since our questionnaire was piloted and tested among a different population, and its psychometric properties remains to be tested, it is unclear how the respondents' answers accurately reflect their clinical practice. Our results might not generalize to chiropractors outside of Quebec since the training and context of practice might differ.

## Conclusion

Vital signs and cranial nerve examinations were the most frequently reported non-musculoskeletal examinations by chiropractors. Apart from the genitourinary exam almost never performed, most participants chose non-musculoskeletal examinations deemed appropriate for the patient’s presentation. The main reason for not performing a specific physical examination was the participants’ belief that another healthcare provider should perform it.

### Supplementary Information

**Additional file 1:** Survey sent to participants.

## Data Availability

The datasets analysed during the current study are available from the corresponding author on reasonable request.

## Abbreviations

ANOVA: Analyze of variance
CMCC: Canadian Memorial Chiropractic College
HBP: High blood pressure
LBP: Low back pain
Non-MSK: Non-musculoskeletal
OCQ: Ordre des Chiropraticiens du Québec
SD: Standard deviation
UQTR: Université du Québec à Trois-Rivières
