# Supplementary material for: Frequency and indication of non-musculoskeletal examinations: a cross-sectional survey of Quebec chiropractors
Source: Chiropr Man Therap. 2024 Feb 28;32:6. doi: 10.1186/s12998-023-00522-z (PMC10903024; doi:10.1186/s12998-023-00522-z)
Supplement: Supplementary file 1 — Additional file 1: Survey sent to participants. [file 12998_2023_522_MOESM1_ESM.pdf]

Fréquence et indication des examens non-musculosquelettiques chez les chiropraticien(ne)s

Nom :_______________________________

Code permanent : ____________________

SECTION 1 / 3 : VOLET INFORMATIONS GÉNÉRALES

1. Êtes-vous :

- ☐ Un homme
- ☐ Une femme
- ☐ Autre
- ☐ Préfère ne pas répondre

2. Dans quelle institution d'enseignement avez-vous complété votre formation en chiropratique?

- ☐ UQTR
- ☐ CMCC
- ☐ Autre

3. Avez-vous suivi une formation post-graduée en chiropratique, et si oui, laquelle?

- ☐ Aucune
- ☐ Maîtrise (MSc ou autres) et/ou doctorat de troisième cycle (PhD)
- ☐ Spécialisation chiropratique de type fellow ou Diplomate

4. Actuellement, combien d'heures par semaine pratiquez-vous en moyenne la chiropratique?

Mots : Caractères : / 4000

5. Depuis combien d'années pratiquez-vous la chiropratique?

Mots : Caractères : / 4000

6. Êtes-vous actuellement employé dans une institution canadienne d'enseignement de la chiropratique (UQTR ou CMCC)? *Cochez le ou les énoncé(s) qui s'applique(nt)*

- [ ] Non
- [ ] Chargé de cours
- [ ] Professeur (régulier ou clinicien)
- [ ] Clinicien à la clinique universitaire

SECTION 2 / 3 : VOLET FRÉQUENCE

Dans une année de pratique typique, à quelle fréquence exécutez-vous en moyenne les manœuvres d'examen physique suivantes chez un patient? Cochez l'énoncé le plus juste.

		**Au moins une fois par jour**	**Au moins une fois par semaine**	**Au moins une fois par mois**	**Au moins une fois par trimestre**	**Au moins une fois par année**	**Jamais**
**1.**	Pression artérielle	☐ Au moins une fois par jour	☐ Au moins une fois par semaine	☐ Au moins une fois par mois	☐ Au moins une fois par trimestre	☐ Au moins une fois par année	☐ Jamais
**2.**	Fréquence cardiaque	☐ Au moins une fois par jour	☐ Au moins une fois par semaine	☐ Au moins une fois par mois	☐ Au moins une fois par trimestre	☐ Au moins une fois par année	☐ Jamais
**3.**	Fréquence respiratoire	☐ Au moins une fois par jour	☐ Au moins une fois par semaine	☐ Au moins une fois par mois	☐ Au moins une fois par trimestre	☐ Au moins une fois par année	☐ Jamais
**4.**	Température	☐ Au moins une fois par jour	☐ Au moins une fois par semaine	☐ Au moins une fois par mois	☐ Au moins une fois par trimestre	☐ Au moins une fois par année	☐ Jamais
**5.**	Saturation en oxygène	☐ Au moins une fois par jour	☐ Au moins une fois par semaine	☐ Au moins une fois par mois	☐ Au moins une fois par trimestre	☐ Au moins une fois par année	☐ Jamais

6. Examen neurologique

		Au moins une fois par jour	Au moins une fois par semaine	Au moins une fois par mois	Au moins une fois par trimestre	Au moins une fois par année	Jamais
**7.**	Examen des nerfs crâniens	☐ Au moins une fois par jour	☐ Au moins une fois par semaine	☐ Au moins une fois par mois	☐ Au moins une fois par trimestre	☐ Au moins une fois par année	☐ Jamais
**8.**	Manoeuvres vestibulaires (i.e. Dix-Hallpike et/ou Fukuda et/ou brassage de tête)	☐ Au moins une fois par jour	☐ Au moins une fois par semaine	☐ Au moins une fois par mois	☐ Au moins une fois par trimestre	☐ Au moins une fois par année	☐ Jamais
**9.**	Épreuves cérébelleuses (i.e. recherche d'un nystagmus et/ou d'adiadococinésie et/ou de dysmétrie et/ou de dysarthrie et/ou d'hypotonie et/ou d'ataxie)	☐ Au moins une fois par jour	☐ Au moins une fois par semaine	☐ Au moins une fois par mois	☐ Au moins une fois par trimestre	☐ Au moins une fois par année	☐ Jamais
**10.**	Signes méningés (i.e. raideur nucale dans le plan sagittal et/ou Soto-Hall et/ou signe de Brudzinski et/ou signe de Kernig)	☐ Au moins une fois par jour	☐ Au moins une fois par semaine	☐ Au moins une fois par mois	☐ Au moins une fois par trimestre	☐ Au moins une fois par année	☐ Jamais

11. Examen ophtalmologique

		Au moins une fois par jour	Au moins une fois par semaine	Au moins une fois par mois	Au moins une fois par trimestre	Au moins une fois par année	Jamais
**12.**	Observation du fond d'oeil (ophtalmoscope)	☐ Au moins une fois par jour	☐ Au moins une fois	☐ Au moins une fois par mois	☐ Au moins une fois	☐ Au moins une fois	☐ Jamais

			par semaine		par trimestre	par année	

13. Examen otorhinolaryngologiste (ORL)

		Au moins une fois par jour	Au moins une fois par semaine	Au moins une fois par mois	Au moins une fois par trimestre	Au moins une fois par année	Jamais
**14.**	Observation du tympan (otoscope)	☐ Au moins une fois par jour	☐ Au moins une fois par semaine	☐ Au moins une fois par mois	☐ Au moins une fois par trimestre	☐ Au moins une fois par année	☐ Jamais

15. Examen pulmonaire

		Au moins une fois par jour	Au moins une fois par semaine	Au moins une fois par mois	Au moins une fois par trimestre	Au moins une fois par année	Jamais
**16.**	Auscultation pulmonaire	☐ Au moins une fois par jour	☐ Au moins une fois par semaine	☐ Au moins une fois par mois	☐ Au moins une fois par trimestre	☐ Au moins une fois par année	☐ Jamais

17. Examen cardiaque

		Au moins une fois par jour	Au moins une fois par semaine	Au moins une fois par mois	Au moins une fois par trimestre	Au moins une fois par année	Jamais
**18.**	Auscultation cardiaque	☐ Au moins une fois par jour	☐ Au moins une fois	☐ Au moins une fois par mois	☐ Au moins une fois	☐ Au moins une fois	☐ Jamais

			par semaine		par trimestre	par année	

19. **Examen vasculaire périphérique**

		Au moins une fois par jour	Au moins une fois par semaine	Au moins une fois par mois	Au moins une fois par trimestre	Au moins une fois par année	Jamais
**20.**	Auscultation des artères carotides et/ou de l'aorte abdominale et/ou des artères rénales et/ou fémorales	☐ Au moins une fois par jour	☐ Au moins une fois par semaine	☐ Au moins une fois par mois	☐ Au moins une fois par trimestre	☐ Au moins une fois par année	☐ Jamais
**21.**	Indice tibio-brachial (i.e. déterminer le rapport entre la pression artérielle mesurée à la cheville et la pression artérielle brachiale)	☐ Au moins une fois par jour	☐ Au moins une fois par semaine	☐ Au moins une fois par mois	☐ Au moins une fois par trimestre	☐ Au moins une fois par année	☐ Jamais
**22.**	Test d'Allen (i.e. compression des artères radiale et ulnaire, puis décompression de l'une des deux artères afin de vérifier le retour vasculaire)	☐ Au moins une fois par jour	☐ Au moins une fois par semaine	☐ Au moins une fois par mois	☐ Au moins une fois par trimestre	☐ Au moins une fois par année	☐ Jamais
**23.**	Signe de Homans (i.e. dorsiflexion de la cheville en vue d'exacerber la douleur au mollet en cas de thrombophlébite)	☐ Au moins une fois par jour	☐ Au moins une fois par semaine	☐ Au moins une fois par mois	☐ Au moins une fois par trimestre	☐ Au moins une fois par année	☐ Jamais
**24.**	Signe du Godet (i.e. persistance d'une empreinte digitale sur un membre due à la présence d'oedème)	☐ Au moins une fois par jour	☐ Au moins une fois par semaine	☐ Au moins une fois par mois	☐ Au moins une fois par trimestre	☐ Au moins une fois par année	☐ Jamais
**25.**	Tests de changement de couleur posturaux tels que le test de Buerger et/ou le test de Tredelenburg (i.e. décoloration et recoloration des membres selon leur position par rapport à la gravité)	☐ Au moins une fois par jour	☐ Au moins une fois	☐ Au moins une fois par mois	☐ Au moins une fois	☐ Au moins une fois	☐ Jamais

			par semaine		par trimestre	par année	
**26.**	Mesure de la circonférence des membres (ex.: mollets)	Au moins une fois par jour	Au moins une fois par semaine	Au moins une fois par mois	Au moins une fois par trimestre	Au moins une fois par année	Jamais

27. **Examen abdominal**

		**Au moins une fois par jour**	**Au moins une fois par semaine**	**Au moins une fois par mois**	**Au moins une fois par trimestre**	**Au moins une fois par année**	**Jamais**
**28.**	Examen général de l'abdomen (i.e. auscultation et/ou percussion et/ou palpation superficielle et/ou palpation profonde)	Au moins une fois par jour	Au moins une fois par semaine	Au moins une fois par mois	Au moins une fois par trimestre	Au moins une fois par année	Jamais
**29.**	Palpation spécifique des organes abdominaux (i.e. palpation de l'aorte abdominale et/ou du foie et/ou des reins et/ou de la rate	Au moins une fois par jour	Au moins une fois par semaine	Au moins une fois par mois	Au moins une fois par trimestre	Au moins une fois par année	Jamais
**30.**	Manoeuvres spéciales (i.e. signe de Murphy et/ou point de McBurny et/ou test de l'onde liquide et/ou signe du rebond et/ou punch rénal et/ou signe du psoas et/ou signe de l'obturateur et/ou signe d'appréhension et/ou signe de Rovsing)	Au moins une fois par jour	Au moins une fois par semaine	Au moins une fois par mois	Au moins une fois par trimestre	Au moins une fois par année	Jamais

31. **Examen des seins**

		**Au moins une fois par jour**	**Au moins une fois par semaine**	**Au moins une fois par mois**	**Au moins une fois par trimestre**	**Au moins une fois par année**	**Jamais**
**32.**	Examen des seins (i.e. palpation des seins à la recherche de	Au	Au	Au	Au	Au	

	masses au niveau du tissu mammaire)	moins une fois par jour	moins une fois par semaine	moins une fois par mois	moins une fois par trimestre	moins une fois par année	Jamais

33. Examen génito-urinaire

		**Au moins une fois par jour**	**Au moins une fois par semaine**	**Au moins une fois par mois**	**Au moins une fois par trimestre**	**Au moins une fois par année**	**Jamais**
**34.**	Toucher rectal	☐ Au moins une fois par jour	☐ Au moins une fois par semaine	☐ Au moins une fois par mois	☐ Au moins une fois par trimestre	☐ Au moins une fois par année	☐ Jamais
**35.**	Toucher vaginal	☐ Au moins une fois par jour	☐ Au moins une fois par semaine	☐ Au moins une fois par mois	☐ Au moins une fois par trimestre	☐ Au moins une fois par année	☐ Jamais
**36.**	Examen des testicules (i.e. palpation des testicules à la recherche de masses)	☐ Au moins une fois par jour	☐ Au moins une fois par semaine	☐ Au moins une fois par mois	☐ Au moins une fois par trimestre	☐ Au moins une fois par année	☐ Jamais
**37.**	Examen du canal inguinal (i.e. recherche de protrusion ou de masse inguinale en position couchée et/ou debout)	☐ Au moins une fois par jour	☐ Au moins une fois par semaine	☐ Au moins une fois par mois	☐ Au moins une fois par trimestre	☐ Au moins une fois par année	☐ Jamais

SECTION 3 / 3 : VOLET INDICATION

La section suivante comprend sept (7) courtes vignettes cliniques décrivant la présentation de patients fictifs. Prenez d'abord connaissance des cas qui vous sont présentés et sélectionnez ensuite les examens physiques non-musculosquelettiques que vous jugez pertinents en vue d'établir un diagnostic différentiel juste selon la présentation clinique, comme vous le feriez en clinique.

1. Monsieur Richard, un électricien de 45 ans, se présente à votre clinique avec une douleur lombaire similaire aux autres épisodes précédents. En effet, vous avez traité ce monsieur à deux reprises dans les 4 dernières années pour un syndrome sacro-iliaque. Cette fois-ci, il dit que sa douleur s'est exacerbée beaucoup plus rapidement et est plus intense qu'à l'habitude. Il vous confie de façon un peu timide qu'il a l'impression que la douleur irradie dans la région inguinale et testiculaire gauche. Bien qu'il reporte être en bonne santé générale, il avoue se sentir un peu moche ce soir.

Quel(s) examen(s) physique(s) non-musculosquelettique(s) jugez-vous pertinent(s) d'exécuter chez ce patient?

- ☐ Pression artérielle
- ☐ Fréquence cardiaque
- ☐ Fréquence respiratoire
- ☐ Température
- ☐ Saturation en oxygène
- ☐ Examen des nerfs crâniens
- ☐ Manoeuvres vestibulaires
- ☐ Épreuves cérébelleuses
- ☐ Signes méningés
- ☐ Observation du fond d'oeil
- ☐ Observation du tympan
- ☐ Auscultation pulmonaire
- ☐ Auscultation cardiaque
- ☐ Auscultation des artères carotides et/ou de l'aorte abdominale et/ou des artères rénales et/ou fémorales
- ☐ Indice tibio-brachial
- ☐ Test d'Allen
- ☐ Signe de Homans
- ☐ Signe du Godet
- ☐ Tests de changement de couleur posturaux (ex.: Buerger)
- ☐ Mesure de la circonférence des membres
- ☐ Examen général de l'abdomen (percussion, auscultation et palpation)
- ☐ Palpation spécifique des organes abdominaux
- ☐ Manoeuvres spéciales abdominales (ex.: Punch rénal, signe du Murphy, point de McBurny, etc)
- ☐ Examen des seins

- ☐ Toucher rectal
- ☐ Toucher vaginal
- ☐ Examen des testicules
- ☐ Examen du canal inguinal

---

2. Monsieur Desmarais, un homme âgé de 65 ans, fume depuis plus de 40 ans et il souffre d'hypertension artérielle, d'hypercholestérolémie et d'embonpoint. Il vous a consulté à plusieurs reprises par le passé pour une lombalgie mécanique (non-spécifique) récurrente. Il se présente à votre clinique pour une douleur lombaire basse et diffuse ayant débuté la veille. Cependant, vous êtes incapable de reproduire la plainte du patient à la palpation ni à l'examen orthopédique cette fois.

Quel(s) examen(s) physique(s) non-musculosquelettique(s) jugez-vous pertinent(s) d'exécuter chez ce patient?

- ☐ Pression artérielle
- ☐ Fréquence cardiaque
- ☐ Fréquence respiratoire
- ☐ Température
- ☐ Saturation en oxygène
- ☐ Examen des nerfs crâniens
- ☐ Manoeuvres vestibulaires
- ☐ Épreuves cérébelleuses
- ☐ Signes méningés
- ☐ Observation du fond d'oeil
- ☐ Observation du tympan
- ☐ Auscultation pulmonaire
- ☐ Auscultation cardiaque
- ☐ Auscultation des artères carotides et/ou de l'aorte abdominale et/ou des artères rénales et/ou fémorales
- ☐ Indice tibio-brachial
- ☐ Test d'Allen
- ☐ Signe de Homans
- ☐ Signe du Godet
- ☐ Tests de changement de couleur posturaux (ex.: Buerger)
- ☐ Mesure de la circonférence des membres

- [ ] Examen général de l'abdomen (percussion, auscultation et palpation)
- [ ] Palpation spécifique des organes abdominaux
- [ ] Manoeuvres spéciales abdominales (ex.: Punch rénal, signe du Murphy, point de McBurny, etc)
- [ ] Examen des seins
- [ ] Toucher rectal
- [ ] Toucher vaginal
- [ ] Examen des testicules
- [ ] Examen du canal inguinal

---

3. Monsieur Côté est âgé de 76 ans et se considère actif et en bonne santé générale. Il vous consulte en soins de maintien (de façon préventive) depuis quelques années. Cependant, lors de sa visite cet après-midi, il vous mentionne qu’il ressent une importante douleur à la poitrine à droite depuis ce matin. La douleur est survenue alors qu’il pelletait son entrée. La douleur est soulagée par le repos et exacerbée par l’activité. Vous êtes incapables de reproduire précisément la plainte du patient à l’examen physique.

Quel(s) examen(s) physique(s) non-musculosquelettique(s) jugez-vous pertinent(s) d’exécuter chez ce patient?

- [ ] Pression artérielle
- [ ] Fréquence cardiaque
- [ ] Fréquence respiratoire
- [ ] Température
- [ ] Saturation en oxygène
- [ ] Examen des nerfs crâniens
- [ ] Manoeuvres vestibulaires
- [ ] Épreuves cérébelleuses
- [ ] Signes méningés
- [ ] Observation du fond d'oeil
- [ ] Observation du tympan
- [ ] Auscultation pulmonaire
- [ ] Auscultation cardiaque
- [ ] Auscultation des artères carotides et/ou de l'aorte abdominale et/ou des artères rénales et/ou fémorales
- [ ] Indice tibio-brachial
- [ ] Test d'Allen

- [ ] Signe de Homans
- [ ] Signe du Godet
- [ ] Tests de changement de couleur posturaux (ex.: Buerger)
- [ ] Mesure de la circonférence des membres
- [ ] Examen général de l'abdomen (percussion, auscultation et palpation)
- [ ] Palpation spécifique des organes abdominaux
- [ ] Manoeuvres spéciales abdominales (ex.: Punch rénal, signe du Murphy, point de McBurny, etc)
- [ ] Examen des seins
- [ ] Toucher rectal
- [ ] Toucher vaginal
- [ ] Examen des testicules
- [ ] Examen du canal inguinal

---

4. Madame Bélanger, une étudiante de 22 ans, se présente à son rendez-vous de suivi régulier cet après-midi. Elle vous confie qu'elle vit une importante période de stress et qu'elle ressent même une douleur thoracique à droite qui a débuté pendant son examen plus tôt dans la journée. Elle semble un peu inquiète car elle vous indique s'être sentie à court de souffle dans les derniers jours. Elle a l'impression que la douleur s'exacerbe chaque fois qu'elle inspire. Elle rapporte par ailleurs être en bonne santé générale et ne prend aucune médication mise à part des contraceptifs oraux.

Quel(s) examen(s) physique(s) non-musculosquelettique(s) jugez-vous pertinent(s) d'exécuter chez cette patiente?

- [ ] Pression artérielle
- [ ] Fréquence cardiaque
- [ ] Fréquence respiratoire
- [ ] Température
- [ ] Saturation en oxygène
- [ ] Examen des nerfs crâniens
- [ ] Manoeuvres vestibulaires
- [ ] Épreuves cérébelleuses
- [ ] Signes méningés
- [ ] Observation du fond d'oeil
- [ ] Observation du tympan
- [ ] Auscultation pulmonaire

- [ ] Auscultation cardiaque
- [ ] Auscultation des artères carotides et/ou de l'aorte abdominale et/ou des artères rénales et/ou fémorales
- [ ] Indice tibio-brachial
- [ ] Test d'Allen
- [ ] Signe de Homans
- [ ] Signe du Godet
- [ ] Tests de changement de couleur posturaux (ex.: Buerger)
- [ ] Mesure de la circonférence des membres
- [ ] Examen général de l'abdomen (percussion, auscultation et palpation)
- [ ] Palpation spécifique des organes abdominaux
- [ ] Manoeuvres spéciales abdominales (ex.: Punch rénal, signe du Murphy, point de McBurny, etc)
- [ ] Examen des seins
- [ ] Toucher rectal
- [ ] Toucher vaginal
- [ ] Examen des testicules
- [ ] Examen du canal inguinal

---

5. Madame Bolduc, une technicienne de laboratoire de 26 ans vous consulte depuis quelques semaines pour des cervicalgies associées à des tensions musculaires. Lors de sa visite aujourd’hui, elle vous raconte que lors d’une partie de volleyball la veille, elle a reçu un ballon en plein visage, sur son œil gauche après une puissante attaque (“smash”) de son ami. Lorsque vous la questionnez, elle vous indique qu’elle n’a pas ressenti de maux de tête ou de nausées depuis, mais elle a l’impression qu’elle voit moins bien. Elle explique avoir l’impression d’avoir des petites taches flottantes dans son champ de vision.

Quel(s) examen(s) physique(s) non-musculosquelettique(s) jugez-vous pertinent(s) d’exécuter chez cette patiente?

- [ ] Pression artérielle
- [ ] Fréquence cardiaque
- [ ] Fréquence respiratoire
- [ ] Température
- [ ] Saturation en oxygène
- [ ] Examen des nerfs crâniens
- [ ] Manoeuvres vestibulaires

- ☐ Épreuves cérébelleuses
- ☐ Signes méningés
- ☐ Observation du fond d'oeil
- ☐ Observation du tympan
- ☐ Auscultation pulmonaire
- ☐ Auscultation cardiaque
- ☐ Auscultation des artères carotides et/ou de l'aorte abdominale et/ou des artères rénales et/ou fémorales
- ☐ Indice tibio-brachial
- ☐ Test d'Allen
- ☐ Signe de Homans
- ☐ Signe du Godet
- ☐ Tests de changement de couleur posturaux (ex.: Buerger)
- ☐ Mesure de la circonférence des membres
- ☐ Examen général de l'abdomen (percussion, auscultation et palpation)
- ☐ Palpation spécifique des organes abdominaux
- ☐ Manoeuvres spéciales abdominales (ex.: Punch rénal, signe du Murphy, point de McBurny, etc)
- ☐ Examen des seins
- ☐ Toucher rectal
- ☐ Toucher vaginal
- ☐ Examen des testicules
- ☐ Examen du canal inguinal

---

6. Madame Charbonneau, une ingénieure de 43 ans, vous consulte pour une cervicalgie présente depuis cinq jours. La douleur l'empêche de tourner la tête. Elle est pire au réveil et est soulagée par la chaleur. La patiente vous indique que la douleur a débuté du côté gauche et que, ce matin, elle la ressentait maintenant du côté droit seulement. Vous la questionnez et elle affirme qu'elle n'a pas ressenti de maux de tête ou d'étourdissement depuis l'apparition de sa cervicalgie. Vous observez la patiente et elle vous semble fatiguée. Elle vous mentionne qu'effectivement, la douleur l'empêche de bien dormir la nuit. Elle rapporte être en bonne santé générale, mais est suivie depuis 5 ans pour de l'hypothyroïdie pour laquelle elle est médicamentée.

Quel(s) examen(s) physique(s) non-musculosquelettique(s) jugez-vous pertinent(s) d'exécuter chez cette patiente?

- ☐ Pression artérielle
- ☐ Fréquence cardiaque

- [ ] Fréquence respiratoire
- [ ] Température
- [ ] Saturation en oxygène
- [ ] Examen des nerfs crâniens
- [ ] Manoeuvres vestibulaires
- [ ] Épreuves cérébelleuses
- [ ] Signes méningés
- [ ] Observation du fond d'oeil
- [ ] Observation du tympan
- [ ] Auscultation pulmonaire
- [ ] Auscultation cardiaque
- [ ] Auscultation des artères carotides et/ou de l'aorte abdominale et/ou des artères rénales et/ou fémorales
- [ ] Indice tibio-brachial
- [ ] Test d'Allen
- [ ] Signe de Homans
- [ ] Signe du Godet
- [ ] Tests de changement de couleur posturaux (ex.: Buerger)
- [ ] Mesure de la circonférence des membres
- [ ] Examen général de l'abdomen (percussion, auscultation et palpation)
- [ ] Palpation spécifique des organes abdominaux
- [ ] Manoeuvres spéciales abdominales (ex.: Punch rénal, signe du Murphy, point de McBurny, etc)
- [ ] Examen des seins
- [ ] Toucher rectal
- [ ] Toucher vaginal
- [ ] Examen des testicules
- [ ] Examen du canal inguinal

---

7. Monsieur Coulombe est un retraité de 68 ans qui souffre d'embonpoint et a recommencé à fumer depuis quelques années. Il se présente à votre clinique pour des céphalées au réveil qui se présentent chaque matin depuis environ un mois. La douleur tend à augmenter légèrement au fil des semaines. Il décide de vous consulter aujourd'hui, car il recense quelques épisodes de vertiges depuis les derniers jours. Celui-ci craint que cela ait un lien avec ses maux de tête. Il ajoute que depuis l'apparition des vertiges, il a l'impression qu'il

entend moins bien du côté gauche et vous décrit également une sensation de plénitude dans son oreille. Celui-ci n'a pas éprouvé de nausée ou de vomissements.

Quel(s) examen(s) physique(s) non-musculosquelettique(s) jugez-vous pertinent(s) d'exécuter chez ce patient?

- [ ] Pression artérielle
- [ ] Fréquence cardiaque
- [ ] Fréquence respiratoire
- [ ] Température
- [ ] Saturation en oxygène
- [ ] Examen des nerfs crâniens
- [ ] Manoeuvres vestibulaires
- [ ] Épreuves cérébelleuses
- [ ] Signes méningés
- [ ] Observation du fond d'oeil
- [ ] Observation du tympan
- [ ] Auscultation pulmonaire
- [ ] Auscultation cardiaque
- [ ] Auscultation des artères carotides et/ou de l'aorte abdominale et/ou des artères rénales et/ou fémorales
- [ ] Indice tibio-brachial
- [ ] Test d'Allen
- [ ] Signe de Homans
- [ ] Signe du Godet
- [ ] Tests de changement de couleur posturaux (ex.: Buerger)
- [ ] Mesure de la circonférence des membres
- [ ] Examen général de l'abdomen (percussion, auscultation et palpation)
- [ ] Palpation spécifique des organes abdominaux
- [ ] Manoeuvres spéciales abdominales (ex.: Punch rénal, signe du Murphy, point de McBurny, etc)
- [ ] Examen des seins
- [ ] Toucher rectal
- [ ] Toucher vaginal
- [ ] Examen des testicules

- [ ] Examen du canal inguinal

8. Estimez le pourcentage des nouveaux patients se présentant à votre clinique chez qui un examen physique non-musculosquelettique serait indiqué?

Mots : Caractères : / 4000

9. Dans quelle proportion de ces nouveaux patients exécutez-vous un examen non-musculosquelettique?

Mots : Caractères : / 4000

10. Dans les cas où vous n'effectuez pas l'examen non-musculosquelettique d'un patient qui nécessiterait un examen non-musculosquelettique, pour quelle(s) raison(s) choisissez-vous de ne pas exécuter cet examen?

- [ ] Je considère que je ne tirerai aucune information pertinente à ma conduite clinique de cet examen
- [ ] Je considère qu'exécuter et/ou interpréter des examens physiques non-musculosquelettiques dépasse mes compétences actuelles (i.e. j'ai gradué il y a longtemps et/ou je ne me souviens plus et/ou je n'ai pas appris à faire ces manoeuvres d'examen physique
- [ ] Je considère qu'un autre professionnel de la santé sera en meilleure position pour exécuter et/ou interpréter l'information tirée de cet examen physique qu'un chiropraticien
- [ ] Je considère que les examens non-musculosquelettiques sont hors de mon champ d'exercice
- [ ] Je n'ai pas le temps
- [ ] Autre :

11. Merci d'avoir participé à ce projet de recherche. Si vous désirez que l'on retire vos coordonnées de notre liste de rappel, veuillez simplement indiquer votre nom et votre adresse courriel ci-dessous.

Mots : Caractères : / 4000
